# Effects of *Clostridium butyricum* on growth performance, meat quality, and intestinal health of broilers

**DOI:** 10.3389/fvets.2023.1107798

**Published:** 2023-01-24

**Authors:** Zhen Li, Lingbo Long, Xu Jin, Yang Li, Qiong Wu, Xingyong Chen, Zhaoyu Geng, Cheng Zhang

**Affiliations:** Department of Animal Science, College of Animal Science and Technology, Anhui Agricultural University, Hefei, China

**Keywords:** broilers, *Clostridium butyricum*, growth performance, meat quality, intestinal health

## Abstract

This study investigated the effects of *Clostridium butyricum* on the growth performance, meat quality and intestinal health of broilers. A total of 800 one-day-old male Arbor Acres broilers were randomly assigned to two groups with 16 replicates of 25 broilers per group and fed with a basal diet (CON) or a basal diet supplemented with 1.5 × 10^9^ cfu/kg *C. butyricum* and 5 × 10^8^ cfu/kg *C. butyricum* at 1–21 d and 22–42 d, respectively (CB). The results indicated that *C. butyricum* significantly increased the final body weight, average daily gain at 1–42 d in the growth performance of broilers (*P* < 0.05). Moreover, *C. butyricum* significantly increased ${\text{a}}_{24\text{h}}^{*}$ value and pH_24h_ value of breast meat but reduced the drip loss and shear force (*P* < 0.05). Regarding serum antioxidant indices, *C. butyricum* significantly increased the total superoxide dismutase (T-SOD) and total antioxidative capacity activities and reduced the malondialdehyde content (*P* < 0.05). Furthermore, the broilers in the CB demonstrated an increase in jejunal lipase and trypsin activities, villus height (VH) and VH-to-crypt depth ratio at 42 d compared with those in the CON (*P* < 0.05). *C. butyricum* also upregulated the intestinal mRNA levels of zonula occludens-1, nuclear factor erythroid 2-related factor 2 *(Nrf2*), *SOD1* and interleukin-10 in the jejunal mucosa (*P* < 0.05), but it downregulated the mRNA levels of nuclear factor kappa B (*NF-*κ*B*) and tumor necrosis factor-α (*P* < 0.05). These results indicate that *C. butyricum* can improve the growth performance and meat quality of broilers. In particular, *C. butyricum* can improve the intestinal health of broilers, which is likely to be related to the activation of the Nrf2 signaling pathway and inhibition of the NF-κB signaling pathway.

## 1. Introduction

Generally, the growth performance of broilers is highly dependent on their intestinal health, which is influenced by a variety of factors, such as diet, stress and infection. These factors may increase the intestinal permeability of broilers and compromise the structural integrity of their intestinal epithelium, resulting in an increase in inflammatory responses and ultimately the restriction of normal growth (1). In recent years, because of the ban on antibiotics in poultry feed, unspecified intestinal diseases have emerged in poultry production (2). Therefore, new alternatives are urgently required to reduce the incidence of intestinal disease and increase the production efficiency of broilers. Among these potential alternatives, probiotics, owing to their ecofriendly and pollution-free characteristics, are typically fed directly to inhibit the growth of pathogens and regulate their intestinal function of broilers (2, 3). Studies have demonstrated that probiotics could prevent pathogenic bacteria from adhering to the intestinal mucosa and hence improve the microecological environment of the gastrointestinal tract in order to optimize the intestinal function of broilers, thereby enhancing their growth performance (4, 5). Hence, dietary supplementation with probiotics is regarded as an effective method for enhancing the growth performance and enteric diseases resistance of broilers.

*Clostridium butyricum CBM 588* is a typical probiotic strain that produces spores that are resistant to high temperature, humidity, stomach acid and bile salts, indicating its high adaptability to the intestinal environment (6). *C. butyricum* metabolites contain digestive enzymes that can break down large molecules of nutrients into small molecules that can be easily absorbed, thereby enhancing the utilization efficiency of these nutrient substances and ultimately promoting the growth performance of the host (6). As a source of energy, short-chain fatty acids (SCFA), another metabolite of *C. butyricum*, not only retard the growth of pathogenic enteric bacteria, but also promote intestinal development. Studies have indicated that the addition of *C. butyricum* to the diet of broilers could increase their intestinal digestive capacity and improve their intestinal morphology, which in turn enhances their growth performance (7, 8). Liao et al. (9) reported that *C. butyricum* treatment increased the concentrations of polyunsaturated fatty acids in the meat and improved the meat quality of broilers. In addition, *C. butyricum* was found to positively affect the immune response of broilers, which could promote the secretion of anti-inflammatory factors and immunoglobulins (10, 11). A recent study on Pekin ducks revealed that dietary *C. butyricum* supplementation increased the SCFA content in the gut and optimized the intestinal flora (12). Although the effects of *C. butyricum* treatment on the growth performance and intestinal health have been extensively documented in the literature on poultry production, the results remain inconsistent. Therefore, the further research on more targeted indicators is necessary to accurately evaluate the benefits of *C. butyricum* and explore the mechanism of through which *C. butyricum* regulates intestinal health. In addition, because of *C. butyricum* and its metabolites showing certain immuno–antioxidant properties in poultry (9, 13), we hypothesize that *C. butyricum* could regulate the nuclear factor erythroid 2-related factor 2 (Nrf2) signaling pathway (a key signaling pathway for regulating antioxidant capacity) and nuclear factor-κB (NF-κB) signaling pathway (a key signaling pathway for regulating inflammatory response). Accordingly, the present study investigated the effects of *C. butyricum* on the growth performance, meat quality and intestinal health of broilers to elucidate the potential mechanism though which *C. butyricum* regulates intestinal health.

## 2. Materials and methods

### 2.1. Experimental design and diets

The experimental procedures performed on the animals included in this study were approved by the Animal Ethics Committee of Anhui Agricultural University. A total of 800 one-day-old male Arbor Acres (AA) broilers from the same batch were randomly assigned to two groups with 16 replicates of 25 broilers per group and fed with a basal diet (CON) or a basal diet supplemented with 1.5 × 10^9^ cfu/kg *C. butyricum* and 5 × 10^8^ cfu/kg *C. butyricum* at 1–21 d and 22–42 d, respectively (CB). The *C. butyricum* additive contained at least 5 × 10^9^ cfu/g powder. The addition level of *C. butyricum* used in this study was based on an unpublished gradient addition trial which found that *C. butyricum* supplementation at 1.5 × 10^9^ cfu/kg from 1 to 21 d and 5 × 10^8^ cfu/kg from 22 to 42 d could maximize the growth performance of broilers. The broilers were provided with mash feed and fresh water *ad libitum* from 1 d until 42 d. The temperature was gradually reduced from 34°C ± 1°C to 23°C ± 1°C at a rate of 2°C or 3°C per week and then maintained at 23°C ± 1°C until the end of the experiment, and the lighting program was set to produce 1 h of darkness and 23 h of light. The coop was longitudinally ventilated using an exhaust fan, and the humidity maintained between 55 and 65%. The basal diets are formulated according to the requirements for broilers by NRC (1994), and their composition and nutritional level are displayed in Supplementary Table 1. Body weight (BW) at 21 d and 42 d and daily feed consumption were documented on a replicate basis to calculate the average daily BW gain (ADG), average daily feed intake (ADFI) and feed to gain ratio (F/G). These indexes of growth performance were calculated using the following formulas.


$$\begin{array}{l} \text{ ADFI=}\Sigma [\left(\text{Feed amount-Residual amount}\right)\\ \text{ /Number of broilers}]\text{/Days}\\ \text{ADG=}(\text{Final average weight-Initial average weight})\text{/Days}\\ \text{ F/G=Total feed consumption/Total weight gain} \end{array}$$

### 2.2. Sample collection

At 21 and 42 d, one broiler of the average pen BW was selected from each replicate and euthanized by electrical stunning, and the remaining broilers meeting the market requirement were sold after the end of the experiment. Blood samples were then collected from the wing vein after 12 h of fasting, placed in polypropylene tubes, and centrifuged at 1500 × *g* for 15 min at 4°C to obtain the serum, which was then stored at −20°C until the analysis of antioxidant-related indicators. After the slaughter and dissection processes, the right breast muscle was removed for the analysis of cooking loss and shear force, and the left breast muscle was removed and kept at 4°C for the analysis of pH, meat color and drip loss. To evaluate the digestive enzyme activity, duodenal, jejunal, and ileal digesta were collected in sterile centrifuge tubes, speedily removed in liquid nitrogen and kept at −80°C. Subsequently, segments measuring 2 cm were cut from the middle of the duodenum, jejunum and ileum and fixed in 10% buffered formalin for morphological evaluation. Mucosal samples were then obtained by gently scraping the jejunal wall using sterile slides, immediately frozen in liquid nitrogen, and stored at −80°C until gene expression analysis.

### 2.3. Meat quality

The color (L^*^, lightness; a^*^, redness; b^*^, yellowness) and pH of breast muscle were measured in triplicate using a chroma meter (CR-300, Minolta Camera, Osaka, Japan) and a pH meter (pH-STAR, SFK-Technology, Copenhagen, Denmark) at 45 min and 24 h postmortem, respectively. Subsequently, drip loss, cooking loss, and shear force were measured using our previously described methods (14).

### 2.4. Metabolite contents and enzyme activities

The intestinal contents were first homogenized in precooled phosphate-buffered saline and centrifuged at 1000 × *g* for 10 min at 4°C, and then the supernatants were obtained. Subsequently, malondialdehyde (MDA) level and the activities of catalase (CAT), total antioxidant capacity (T-AOC), total superoxide dismutase (T-SOD), glutathione peroxidase (GSH-Px) in serum, as well as intestinal amylase, lipase, and trypsin activities were determined by commercially available kits (Nanjing Jiancheng Biochemistry Institute, Nanjing, China) according to the manufacturer's instructions.

### 2.5. Intestinal morphology

Intestinal samples were dehydrated, embedded in paraffin, and stained with hematoxylin and eosin. Subsequently, under an inverted fluorescence microscope, villus height (VH) and crypt depth (CD) were measured and recorded using an image processing and analysis system (Leica Imaging Systems, Cambridge, UK) to evaluate the ratio of VH to CD (VH/CD).

### 2.6. Quantitative reverse transcription PCR

Total RNA was separated from jejunal mucosa using TRIzol (Yeasen, Shanghai, China). The RNA samples were then reverse-transcribed into cDNA for next analysis using a Hifair^®^ II 1st Strand cDNA Synthesis Kit (Yeasen, Shanghai, China), and gene expression levels were quantified using real-time PCR with a Hieff^®^ qPCR SYBR Green Master Mix (Yeasen, Shanghai, China) and a real-time PCR system (Thermo Fisher Scientific, MA, USA). All primers (Supplementary Table 2) were synthesized by Hefei Qingke Biotechnology Co., Ltd. The reaction conditions and parameter settings followed our previous research (15). The relative expression of target genes was determined using the 2^−ΔΔCt^ method based on the expression of β*-actin*.

### 2.7. Statistical analysis

Data were analyzed using SPSS 18.0 (SPSS, Chicago, IL, USA) and are expressed as mean ± standard error. Student's *t-*test was performed to analyze and compare the CON and CB groups. Statistical significance was set at *P* < 0.05.

## 3. Results

### 3.1. Growth performance

As shown in Table 1, the addition of *C. butyricum* significantly increased the BW and ADG of broilers at 42 d and 1–42 d, respectively (*P* < 0.05), and tended to improve their ADG at 22–42 d (*P* = 0.057). However, *C. butyricum* did not have significant effects on the ADFI or F/G at each stage in either group (*P* > 0.05).

**Table 1:** Effect of *C. butyricum* on the growth performance of broiler chickens.

**Items**	**CON**	**CB**	***P*** **-value**
Initial BW (g)	53.09 ± 0.43	53.78 ± 0.30	0.200
BW at 21 d (g)	724.4 ± 5.62	740.2 ± 7.73	0.109
BW at 42 d (g)	2305 ± 10.71	2365 ± 18.76	0.010
**ADG (g)**			
1–21 d	31.97 ± 0.27	32.69 ± 0.37	0.126
22–42 d	75.25 ± 0.57	77.36 ± 0.90	0.057
1–42 d	53.61 ± 0.26	55.02 ± 0.45	0.012
**ADFI (g)**			
1–21 d	52.40 ± 0.51	53.15 ± 0.60	0.346
22–42 d	141.8 ± 1.31	142.4 ± 1.50	0.779
1–42 d	97.10 ± 0.67	97.76 ± 0.90	0.561
**F/G**			
1–21 d	1.64 ± 0.02	1.63 ± 0.03	0.778
22–42 d	1.89 ± 0.01	1.84 ± 0.02	0.138
1–42 d	1.81 ± 0.01	1.78 ± 0.02	0.139

CON, basal diet; CB, basal diet + Clostridium butyricum. BW, Body weight; ADFI, average daily feed intake; ADG, average daily gain; F/G, feed to gain ratio.

### 3.2. Meat quality

As listed in Table 2, the broilers in the CB group had higher a${}_{24\text{h}}^{*}$ and pH_24h_ values and a lower drip loss and shear force than did those in the CON group (*P* < 0.05). In addition, treatment with *C. butyricum* tended to increase their a${}_{45\text{min}}^{*}$ value (*P* = 0.077).

**Table 2:** Effects of *C. butyricum* on the meat quality of broiler.

**Items**	**CON**	**CB**	***P*** **-value**
L${}_{45\text{min}}^{*}$	46.13 ± 0.89	45.24 ± 1.08	0.528
a${}_{45\text{min}}^{*}$	1.64 ± 0.10	1.98 ± 0.16	0.077
b${}_{45\text{min}}^{*}$	12.00 ± 0.52	11.93 ± 0.41	0.911
L${}_{24\text{h}}^{*}$	53.36 ± 0.39	52.64 ± 0.28	0.156
a${}_{24\text{h}}^{*}$	2.77 ± 0.14	3.48 ± 0.31	0.044
b${}_{24\text{h}}^{*}$	14.13 ± 0.45	13.85 ± 0.28	0.617
pH_45min_	6.21 ± 0.06	6.28 ± 0.03	0.308
pH_24h_	5.70 ± 0.06	5.86 ± 0.04	0.048
Drip loss (%)	2.90 ± 0.11	2.60 ± 0.07	0.045
Shear force (*N*)	27.92 ± 1.12	24.32 ± 1.29	0.049
Cooking loss (%)	25.44 ± 1.37	25.18 ± 1.16	0.887

CON, basal diet; CB, basal diet + Clostridium butyricum. L^*^, lightness; a^*^, redness; b^*^, yellowness.

### 3.3. Serum antioxidant statues

As presented in Table 3, the broilers in the CB group recorded the lower (*P* < 0.05) content of MDA at 42 d and higher (*P* < 0.05) activities of T-AOC at 42 d and T-SOD at d 21 and d 42 than did those in the CON group. Furthermore, *C. butyricum* treatment tended to decrease the MDA content at 21 d (*P* = 0.083). However, no marked difference was observed in the activities of GSH-Px and CAT (*P* > 0.05).

**Table 3:** Effects of *C. butyricum* on serum antioxidant capacity of broilers.

**Age**	**Items**	**CON**	**CB**	***P*** **-value**
21 d	MDA (nmol/mL)	3.45 ± 0.14	3.13 ± 0.10	0.083
	GSH-Px (U/mL)	655.2 ± 24.97	723.8 ± 32.39	0.119
	T-SOD (U/mL)	83.85 ± 2.00	90.90 ± 2.55	0.047
	CAT (U/mL)	36.46 ± 0.71	37.21 ± 0.61	0.440
	T-AOC (U/mL)	0.52 ± 0.02	0.55 ± 0.01	0.101
42 d	MDA (nmol/mL)	4.15 ± 0.12	3.67 ± 0.14	0.021
	GSH-Px (U/mL)	837.2 ± 35.40	864.0 ± 18.54	0.518
	T-SOD (U/mL)	117.2 ± 3.00	128.1 ± 2.41	0.015
	CAT (U/mL)	38.77 ± 0.67	39.30 ± 0.39	0.499
	T-AOC (U/mL)	0.68 ± 0.01	0.71 ± 0.01	0.013

CON, basal diet; CB, basal diet + Clostridium butyricum. CAT, catalase; T-SOD, total superoxide dismutase; GSH-Px, glutathione peroxidase; T-AOC, total antioxidative capacity, MDA: malondialdehyde.

### 3.4. Intestinal morphology

As listed in Table 4, no significant difference was observed in CD between the CON and CB groups (*P* > 0.05). However, the addition of *C. butyricum* significantly increased the jejunal VH and VH/CD at 42 d (*P* < 0.05). Similarly, *C. butyricum* supplementation significantly improved the duodenal VH at 21 d (*P* < 0.05). However, no remarkable influence in ileal morphology was observed between the CON and CB groups (*P* > 0.05).

**Table 4:** Effects of *C. butyricum* on intestinal morphology of broilers.

**Age**	**Items**	**CON**	**CB**	***P-*** **value**
21 d	Duodenum			
	VH (μm)	1279 ± 16.17	1329 ± 11.94	0.023
	CD (μm)	204.3 ± 8.61	192.6 ± 4.34	0.227
	VH/CD	6.52 ± 0.30	6.92 ± 0.13	0.211
	Jejunum			
	VH (μm)	1224 ± 24.15	1246 ± 13.65	0.435
	CD (μm)	170.5 ± 5.11	166.2 ± 3.45	0.502
	VH/CD	7.20 ± 0.10	7.52 ± 0.15	0.090
	Ileum			
	VH (μm)	853.9 ± 7.14	858.3 ± 10.64	0.726
	CD (μm)	135.8 ± 4.92	129.8 ± 4.72	0.395
	VH/CD	6.38 ± 0.23	6.73 ± 0.32	0.375
42 d	Duodenum			
	VH (μm)	1473.3 ± 36.37	1524.3 ± 46.83	0.402
	CD (μm)	244.1 ± 7.69	223.0 ± 3.53	0.119
	VH/CD	6.11 ± 0.28	6.66 ± 0.27	0.174
	Jejunum			
	VH (μm)	1443 ± 14.95	1500 ± 21.05	0.036
	CD (μm)	229.6 ± 10.11	213.1 ± 4.22	0.150
	VH/CD	6.37 ± 0.29	7.05 ± 0.10	0.039
	Ileum			
	VH (μm)	1234 ± 14.52	1245 ± 22.09	0.676
	CD (μm)	212.9 ± 5.73	206.9 ± 6.12	0.487
	VH/CD	5.83 ± 0.15	6.06 ± 0.18	0.335

CON, basal diet; CB, basal diet + Clostridium butyricum. VH, villus height; CD, crypt depth; VH/CD: the ratio of villus height to crypt depth.

### 3.5. Digestive enzyme activity

As displayed in Table 5, no remarkable difference was observed in the digestive enzyme activity in the duodenum or ileum between the CON and CB groups (*P* > 0.05). Compared with the CON group, the CB group demonstrated higher lipase activity in the jejunum at 21 and 42 d (*P* < 0.05). Similarly, *C. butyricum* dramatically increased jejunal trypsin activity at 42 d (*P* < 0.05).

**Table 5:** Effect of *C. butyricum* on digestive enzymes activities of broilers.

**Age**	**Items**	**CON**	**CB**	***P*** **-value**
21 d	Duodenum			
	Amylase (U/mg protein)	0.72 ± 0.12	0.75 ± 0.05	0.784
	Lipase (U/g protein)	37.38 ± 1.82	43.20 ± 3.08	0.141
	Trypsin (U/g protein)	3916 ± 227.4	3986 ± 199.7	0.821
	Jejunum			
	Amylase (U/mg protein)	0.86 ± 0.08	0.94 ± 0.10	0.544
	Lipase (U/g protein)	27.16 ± 1.45	33.87 ± 2.49	0.040
	Trypsin (U/g protein)	4911 ± 691.0	5034 ± 310.1	0.868
	Ileum			
	Amylase (U/mg protein)	1.60 ± 0.17	1.63 ± 0.19	0.905
	Lipase (U/g protein)	35.38 ± 3.94	40.27 ± 4.17	0.413
	Trypsin (U/g protein)	7607 ± 241.1	7744 ± 301.4	0.729
42 d	Duodenum			
	Amylase (U/mg protein)	0.85 ± 0.03	0.92 ± 0.04	0.175
	Lipase (U/g protein)	106.0 ± 12.84	118.9 ± 5.60	0.415
	Trypsin (U/g protein)	8956 ± 432.6	9650 ± 737.0	0.501
	Jejunum			
	Amylase (U/mg protein)	1.39 ± 0.09	1.49 ± 0.12	0.498
	Lipase (U/g protein)	100.3 ± 3.90	120.0 ± 7.08	0.048
	Trypsin (U/g protein)	10581 ± 1239	13335 ± 537	0.046
	Ileum			
	Amylase (U/mg protein)	1.73 ± 0.18	1.75 ± 0.12	0.917
	Lipase (U/g protein)	100.2 ± 6.11	110.5 ± 9.34	0.366
	Trypsin (U/g protein)	10060 ± 607.9	11356 ± 460.7	0.112

CON, basal diet; CB, basal diet + Clostridium butyricum.

### 3.6. mRNA expression levels of intestinal barrier genes

**Figure 1:**
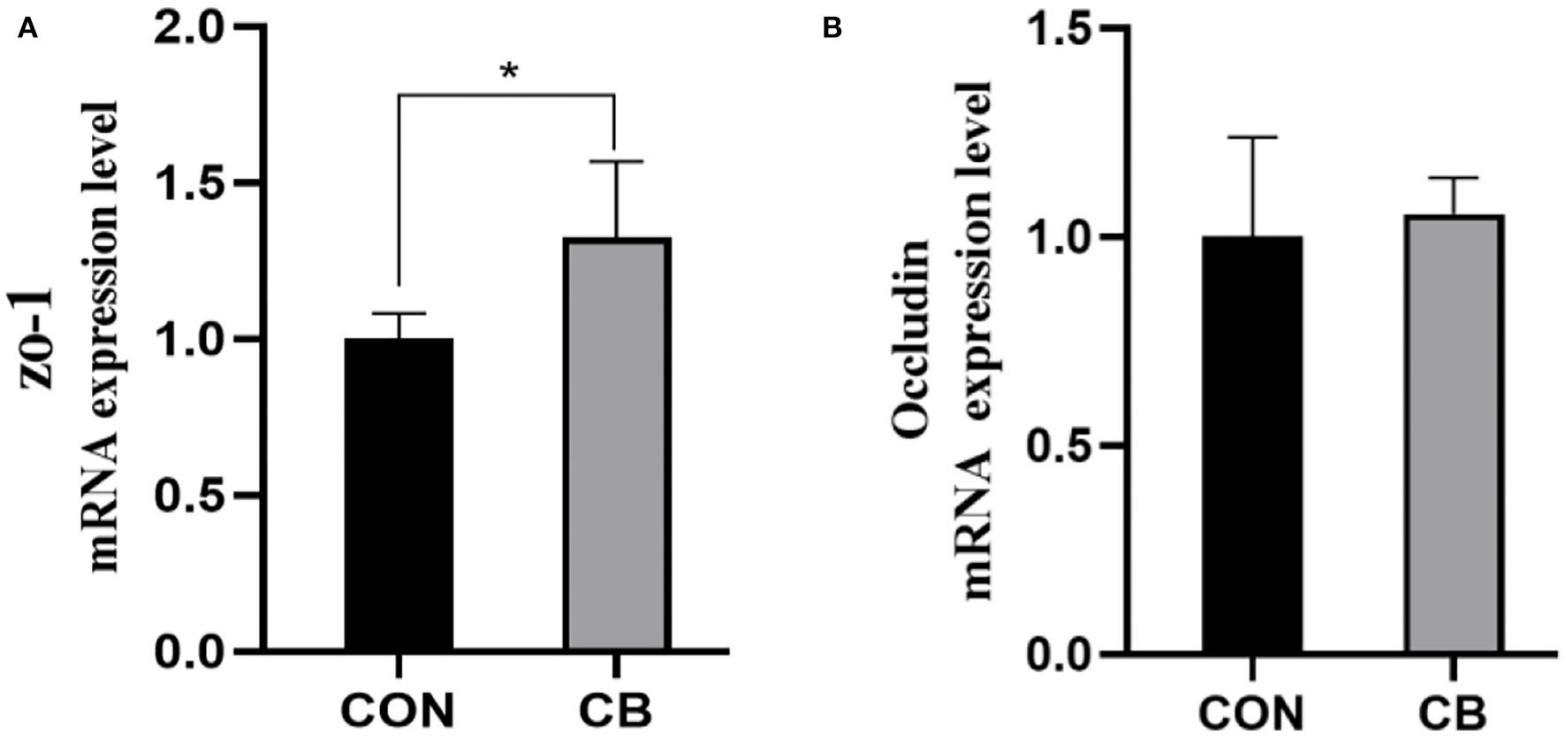
**(A, B)** Effect of *C. butyricum* on the mRNA expression levels of jejunal barrier genes of broilers. CON, basal diet; CB, basal diet + *Clostridium butyricum*. ^*^Significant difference between group (*P* < 0.05). *ZO-1*, zonula occludin protein 1.

As illustrated in Figure 1, the CB group had a higher expression level of the tight-junction-protein-related gene *ZO-1* than did the CON group (*P* < 0.05). However, occludin level was similar in both groups (*P* > 0.05).

### 3.7. mRNA expression levels of antioxidant-related genes

As presented in Table 6, *C. butyricum* addition effectively increased the jejunal mRNA levels of *Nrf2* and *SOD1* in the broilers (*P* < 0.05). However, no significant differences were observed in the mRNA levels of *CAT* or *GSH-Px* (*P* > 0.05).

**Table 6:** Effect of *C. butyricum* on the mRNA expression levels of jejunal antioxidant-related genes of broilers.

**Items**	**CON**	**CB**	***P*** **-value**
*Nrf2*	1.00 ± 0.08	1.32 ± 0.09	0.031
*CAT*	1.00 ± 0.19	1.12 ± 0.10	0.631
*SOD1*	1.00 ± 0.10	1.37 ± 0.11	0.029
*GSH-Px*	1.00 ± 0.18	1.10 ± 0.09	0.621

CON, basal diet; CB, basal diet + Clostridium butyricum. Nrf2, nuclear factor erythroid 2-related factor 2; CAT, catalase; SOD1, superoxide dismutase 1; GSH-Px, glutathione peroxidase.

### 3.8. mRNA expression levels of inflammation-related genes

As listed in Table 7, *C. butyricum* effectively downregulated jejunal mRNA expression of *NF-*κ*B* and tumor necrosis factor-α (*TNF-*α) (*P* < 0.05), but it upregulated interleukin (*IL*)*-10* mRNA expression (*P* < 0.05). However, no significant difference was observed in the mRNA expression levels of *IL-1*β or *IL-6* between the CON and CB groups (*P* > 0.05).

**Table 7:** Effect of *C. butyricum* on the mRNA expression levels of jejunal inflammation-related genes of broilers.

**Items**	**CON**	**CB**	***P*** **-value**
*NF-κB*	1.00 ± 0.05	0.81 ± 0.07	0.033
*TNF-α*	1.00 ± 0.07	0.78 ± 0.05	0.027
*IL-1β*	1.00 ± 0.13	0.96 ± 0.05	0.843
*IL-6*	1.00 ± 0.09	0.87 ± 0.04	0.219
*IL-10*	1.00 ± 0.08	1.34 ± 0.11	0.023

CON, basal diet; CB, basal diet + Clostridium butyricum. NF-κB, nuclear factor kappa B; IL-1β, interleukin-1β; IL-6, interleukin-6; IL-10, interleukin-10; TNF-α, tumor necrosis factor-α.

## 4. Discussion

The development of intensive production has predisposed poultry to diseases, which in turn inhibits their growth and development. Including probiotics such as *C. butyricum* in their diets is therefore regarded as an effective solution to this problem (16, 17). As the most reliable indicator of broiler growth and development, growth performance is the key to increase economic efficiency. According to a previous study, AA broilers fed with 1 × 10^9^ cfu/kg *C. butyricum* exhibited an increase in ADG and a decrease in F/G at 1–21 and 1–42 days, respectively (18). Nevertheless, Yang et al. (11) and Liao et al. (16) discovered that different doses of *C. butyricum* addition significantly increased the ADG of broilers at 22–42 d but did not considerably affect their feed conversion ratio. In the present study, we investigated the effect of *C. butyricum* on the growth performance of AA broilers. Our results indicate that although *C. butyricum* did not considerably affect the growth performance of the broilers at 1–21 days, it significantly improved their final BW and ADG at 42 and 1–42 days, respectively, which is consistent with the results of Yang et al. (11). These results suggest that *C. butyricum* treatment improves the growth performance of broilers, which may be due to the production of butyric acid, vitamin B, and amylase by *C. butyricum* to enhance the utilization of feed nutrients (19). In addition, different effects of *C. butyricum* supplementation have been observed in various studies, which could be attributed to some essential factors such as additive dosage, broiler breed and physiological stage, diet composition, and feeding management.

With the rise in living standards, consumers have become increasingly concerned with meat quality, especially in China. Meat quality is an accurate indicator of the physical and chemical properties of meat. In general, L^*^, a^*^, and b^*^ are used to evaluate the color of meat, which is a crucial indicator of the meat's freshness and quality (20). After broilers are slaughtered, the lactic acid is produced by glycolysis accumulates in the muscle tissue, resulting in a decrease in the pH value, protein denaturation, and a decline in the ability of muscle protein to bind water (21). Thus, the final pH and rate of pH decline in meat have a direct effect on the drip loss and ultimately on the water retention capacity and tenderness of the meat. Liu et al. (20) reported that *C. butyricum* treatment increased the pH_45min_ and a^*^ values but reduced the L^*^ value, drip loss and shear force of in Peking duck breast meat. In another study, Li et al. (22) dicovered that Partridge shank chickens fed with a synbiotic diet including *C. butyricum* exhibited lower breast muscle drip loss and cooking loss and higher a${}_{24\text{h}}^{*}$ and pH_24h_ values. In the present study, we discovered that *C. butyricum* increased the a${}_{24\text{h}}^{*}$ and pH_24h_ values but reduced the drip loss and shear force of breast meat, indicating an improvement of the freshness, tenderness and water retention capacity of the meat. These favorable results indicate that *C. butyricum* can enhance the meat quality of broilers.

Antioxidant enzyme activity and oxidation product concentration are reliable biomarkers for assessing the antioxidant status of animals. T-SOD is a crucial component of the enzyme system, which effectively scavenges free radicals and inhibits peroxidation. MDA is one of the final peroxidation products derived from membrane lipids. Its level is regarded as a crucial index for quantifying the degree of peroxidation (14). According to previous studies, *C. butyricum* could mitigate peroxidation and improve the antioxidant enzyme activity *in vivo* (13, 16, 23). Li et al. (18) reported that the addition of *C. butyricum* to the diets of broilers enhanced the activities of their serum T-AOC, GSH-Px and SOD. Zhan et al. (13) discovered that *C. butyricum* supplementation resulted in the improvement of T-SOD and GSH?Px activities in the serum of laying hens. Similarly, Han et al. (23) found that *C. butyricum* addition enhanced serum GSH-Px activity in weaned piglets. In the present study, we discovered that *C. butyricum* supplementation significantly decreased the serum MDA content in broilers at 42 d, indicating the lower level of lipid peroxidation. In addition, *C. butyricum* addition significantly increased the activities of T-SOD and T-AOC at 42 d, indicating an improvement of antioxidant capacity. Generally, an improvement in antioxidant capacity may reduce the degree of protein and lipid oxidative damage in muscles and prolong the shelf life of meat (14), further confirming that *C. butyricum* improves the quality of meat.

Digestive enzymes are important for the digestion of nutrient materials and their activities have a substantial effect on the feed conversion and growth performance of broilers. Previous studies have recorded that probiotics can produce digestive enzymes and enhance their activities in the intestine. According to a previous study, Cobb broilers fed with *C. butyricum* exhibited elevated jejunal amylase, lipase, and protease activity (10). Wang and Gu (24) discovered that the addition of *Bacillus coagulans NJ0516* to the diets of broilers resulted in a significant increase in their duodenal protease and amylase activities. However, Rodjan et al. (25) reported that the supplementation of *Bacillus* spores did not significantly affect the digestive enzyme activity of Ross 308 broilers. Similar to the findings of Zhang et al. (10), we found that *C. butyricum* increased the jejunal lipase and trypsin activities in the broilers. These favorable results suggest that *C. butyricum* significantly improves the digestive function of broilers; this is attributable to their metabolism of SCFA, which may stimulate the secretion of digestive enzymes by digestive glands. However, the currently available information is inconsistent and limited, necessitating further evaluation of the underlying mechanism.

The intestine is an essential conduit for the exchange of materials between the external environment and the host environment. Intestinal morphology is a major factor in the evaluation of the intestinal status and is closely linked with the development of the intestinal epithelial structure, which is typically evaluated though the VH, CD and VH/CD. A decrease in CD and an increase in VH and VH/CD indicate a natural increase in epithelial cell turnover and a well-differentiated intestinal mucosa, suggesting an enhanced digestive and absorptive capacity (26). A previous study reported that *C. butyricum* treatment significantly improved the duodenal VH and VH/CD and reduced the duodenal CD in broilers (27). Cao et al. (28) found that broilers fed with *C. butyricum* exhibited an increased VH and a decreased CD in the ileum. Furthermore, a recent study revealed that the addition of *C. butyricum* to the diet of broilers infected with *Clostridium perfringens* increased their intestinal VH/CD and ameliorated the degree of their intestinal damage (29). In the present study, we discovered that *C. butyricum* considerably improved the duodenal VH and jejunal VH and VH/CD of the broilers, indicating an improvement in intestinal morphology. These positive results suggest that *C. butyricum* has a promotive effect on the development of the intestinal epithelial structure and thereby improves the intestinal morphology.

The intestinal barrier is crucial for protecting the host against infection and other diseases and maintaining normal physiological function. In addition to preventing the entry of luminal bacteria and dietary allergens into the mucosa, an intact intestinal barrier selectively regulates the entry of nutrients, ions, and water into the body. ZO-1 and occludin, the main tight junction proteins of the intestinal barrier, play a major role in regulating the integrity and permeability of the gut barrier by binding to the actin cytoskeleton and contributing to the formation of a tight junction structure. According to Li et al. (18), the addition of *C. butyricum* to the diets of broilers increased the relative expression levels of claudin-1 and *ZO-1* in the gut. Liu et al. (30) reported that treatment with *C. butyricum* significantly increased the expression of occludin and *ZO-1* in the jejunum of broilers at 42 d. A similar result was observed in our study; that is, after 42 d of feeding broilers with a diet containing *C. butyricum*, we discovered that the jejunal mRNA levels of *ZO-1* increased, indicating an improvement in the broilers' intestinal barrier integrity.

With the exception of the intestinal barrier, intestinal health is inseparable from intestinal antioxidant status and immune function. As a major regulator, Nrf2 is a basic leucine zipper protein that regulates the expression of genes related to antioxidants or detoxifying enzymes and protects the body against oxidative stress (31, 32). In general, Nrf2 exists in an inactive form in the cytoplasm binding to its inhibitor: Kelch-like epichlorohydrin-related protein 1 (Keap1). In response to oxidative stress, the Keap1–Nrf2 complex dissociates, allowing Nrf2 to translocate into the nucleus and bind to the antioxidant response element. This results in the transcription of antioxidant genes such as heme oxygenase-1, *SOD* and *CAT*. Hence, activating the Nrf2 signaling pathway may increase the expression levels of antioxidant genes (33). In our work, we first found that *C. butyricum* supplementation in broilers resulted in a significant increase in the expression levels of *Nrf2* and *SOD1* in their jejunal mucosa, indicating an improvement in their intestinal antioxidant capacity. This positive finding may be attributable to the activation of the Nrf2 signaling pathway, which effectively protects the intestinal mucosa against oxidative stress damage (33). Moreover, NF-κB, a key transcription factor involved in immunity, can recognize, rapidly respond to, and transcribe a variety of proinflammatory cytokines and inflammatory mediators, such as *TNF-*α and *ILs*, resulting in an inflammatory crisis (34). Reduced expression of genes encoding proinflammatory cytokines, such as interferon-γ, *IL-1*β and *TNF-*α, may retard the activation of the NF-κB signaling pathway. Li et al. (18) demonstrated that *C. butyricum* increased the expression levels of *IL-1*β and *TNF-*α in the ileum of broilers. Besides, a study on weaned piglets challenged with lipopolysaccharides revealed that *C. butyricum* downregulated the expression of *IL-1*β, *TNF-*α and *NF-*κ*B*, thereby mitigating inflammatory damage (35). Similarly, in the present study, we discovered that *C. butyricum* treatment significantly downregulated the mRNA expression of *NF-*κ*B* and *TNF-*α in the jejunal mucosa of the broilers. Moreover, we first observed that *C. butyricum* significantly increased the mRNA expression of *IL-10* in their mucosa. These positive results indicate the lower inflammation and the improved immune status, which is by likely inhibiting NF-κB signaling pathway. Indeed, a potential cross-link exists between the Nrf2 and NF-κB signaling pathways (36), and inhibiting the Nrf2 signaling pathway reduces the antioxidant capacity and increases the likelihood of oxidative stress damage to the body, ultimately leading to inflammation through NF-κB signaling pathway activation (37, 38). Therefore, improvements in intestinal health, including the intestinal morphology, intestinal barrier, antioxidant capacity and immune status, induced by *C. butyricum* may be closely related to the activation of the Nrf2 signaling pathway and inhibition of the NF-κB signaling pathway.

## 5. Conclusions

The addition of *C. butyricum* to the diets of broilers can improve their growth performance and meat quality. *C. butyricum* can also improve intestinal health, which is likely to be linked with the activation of the Nrf2 signaling pathway and inhibition of the NF-κB signaling pathway. However, additional research is necessary to determine the exact mechanism of action of *C. butyricum*.

## Data availability statement

The original contributions presented in the study are included in the article/Supplementary material, further inquiries can be directed to the corresponding author.

## Ethics statement

The animal study was reviewed and approved by the Animal Ethics Committee of Anhui Agricultural University.

## Author contributions

Conceptualization: ZL and XJ. Methodology: LL and YL. Investigation and writing—original draft preparation: ZL and LL. Data curation: QW and YL. Formal analysis: XJ and QW. Writing—review and editing: CZ and XC. Supervision: ZG and CZ. Funding acquisition: CZ. All authors contributed to the article and approved the submitted version.
