# Supplementary material for: Effects of Clostridium butyricum on growth performance, meat quality, and intestinal health of broilers
Source: Front Vet Sci. 2023 Jan 24;10:1107798. doi: 10.3389/fvets.2023.1107798 (PMC9902377; doi:10.3389/fvets.2023.1107798)
Supplement: Supplementary file 1 [file Table_1.DOCX]

Supplementary Material

## Table S1. Composition and nutrient levels of the basal diets

| **Item** | **1-14 d** | **15-28 d** | **29-42 d** |
| --- | --- | --- | --- |
| Ingredients (%) |  |  |  |
| Corn | 59.2 | 58.8 | 60.3 |
| Soybean meal | 27.6 | 31.6 | 30.5 |
| Fish meal | / | 0.8 | 0.8 |
| Corn gluten meal | 7.2 | 2.0 | 2.0 |
| Soybean oil | 1.6 | 2.7 | 2.8 |
| DL-Methionine | 0.21 | 0.25 | 0.14 |
| Lysine-Hydrochloride | 0.30 | 0.16 | 0.11 |
| Sodium chloride | 0.25 | 0.25 | 0.25 |
| Choline chloride | 0.2 | 0.2 | 0.2 |
| Calcium hydrogen phosphate | 1.9 | 1.84 | 1.55 |
| Limestone | 1.30 | 1.16 | 1.11 |
| Premix ^1^ | 0.24 | 0.24 | 0.24 |
| Nutrient level |  |  |  |
| Metabolizable energy (MJ/kg) | 12.56 | 12.51 | 12.61 |
| Crude protein (%) | 21.33 | 20.27 | 19.81 |
| Calcium (%) | 1.02 | 0.99 | 0.91 |
| Available phosphorus (%) | 0.45 | 0.44 | 0.38 |
| Total lysine (%) | 1.19 | 1.17 | 1.11 |
| Total methionine (%) | 0.58 | 0.57 | 0.46 |
| Total methionine + cysteine (%) | 0.92 | 0.90 | 0.79 |

^1^ The premix provided the following per kilogram of diet: vitamin A, 4000 IU; vitamin D_3_, 800 IU; vitamin E 44 IU; vitamin K_3_ 0.5 mg; vitamin B_1_, 3.6 mg; vitamin B_2_ 3.75 mg; vitamin B_6_, 5 mg; vitamin B_12_, 0.015 mg; niacin, 40 mg; biotin, 0.2 mg; pantothenic acid, 12 mg; folic acid, 1.3 mg; Cu, 10 mg as CuSO_4_·5H_2_O; Fe, 80 mg as FeSO4; I, 0.6 mg as KI; Zn, 100 mg as ZnSO_4_; Mn, 80 mg as MnSO_4_; Se, 0.15 mg as Na_2_SeO_3_.

**Table S2.** Primers used for Real-Time PCR.

| **Genes** | **GenBank ID** | **Primer sequences（5'—3'）** |
| --- | --- | --- |
| *ZO-1* | XM_015278975.3 | F: TCCCTAAAGGCGAAGAAGTA |
|  |  | R: CAACAATGCGACGATAAACA |
| *occludin* | NM_205128.1 | F: TCCTCATCGTCATCCTGCTCTG |
|  |  | R: CCATCCGCCACGTTCTTCAC |
| *Nrf2* | NM_205117.1 | F: AGTGACCCAGTCTTCATTTC |
|  |  | R: TCTTCCCAAACTTGCTCTAT |
| *GSH-Px* | NM_001277853.3 | F: GGCAAAGTGCTGCTGGTGGTC |
|  |  | R: TCTCCTCGTTGGTGGCGTTCT |
| *SOD1* | NM_205064.2 | F: AAGGGAGGAGTGGCAGAAGT |
|  |  | R: GCTAAACGAGGTCCAGCATT |
| *CAT* | NM_001031215.2 | F: CTTCCTGGTCTTTCTACATTC |
|  |  | R: ATACGCCATCTGTTCTACCT |
| *NF-κB* | NM_001001472.2 | F: ACTTGGCGATCATTCACGAGG |
|  |  | R: AGCGGAGTCTGGCTGAGGTT |
| *IL-6* | NM_204628.1 | F: GAAATCCCTCCTCGCCAATCT |
|  |  | R: CCTCACGGTCTTCTCCATAAACG |
| *IL-10* | NM_001004414.2 | F: GCTGTCACCGCTTCTTCACC |
|  |  | R: TCCCGTTCTCATCCATCTTCTC |
| *IL-1β* | NM_204524.1 | F: GACCAAACTGCTGCGGAGGC |
|  |  | R: CGAAGGACTGTGAGCGGGTGT |
| *TNF-α* | NM_204267.2 | F: TGTTCTATGACCGCCCAGTT |
|  |  | R: TTCAGAGCATCAACGCAAAA |
| *β-actin* | NM_205518.1 | F: TGCGTGACATCAAGGAGAAG |
|  |  | R: GGACTCCATACCCAAGAAAGAT |

*ZO-1*: zonula occludens-1; *Nrf2*: nuclear factor erythroid 2-related factor 2; *GSH-Px*: glutathione peroxidase; *SOD1*: superoxide dismutase 1; *CAT*: catalase; *NF-κB*: nuclear factor kappa B; IL: interleukin; *TNF-α*: tumor necrosis factor-α.
